# Surfing the Waves of SARS-CoV-2: Analysis of Viral Genome Variants Using an NGS Survey in Verona, Italy

**DOI:** 10.3390/microorganisms12050846

**Published:** 2024-04-24

**Authors:** Emil Tonon, Riccardo Cecchetto, Erica Diani, Nicoletta Medaina, Giona Turri, Anna Lagni, Virginia Lotti, Davide Gibellini

**Affiliations:** 1 Department of Diagnostic and Public Health, Microbiology Section, University of Verona, 37134 Verona, Italy; emil.tonon@studenti.univr.it (E.T.); riccardo.cecchetto@univr.it (R.C.); anna.lagni@univr.it (A.L.); virginia.lotti@univr.it (V.L.); davide.gibellini@univr.it (D.G.); 2 UOC Microbiology Unit, AOUI Verona, 37134 Verona, Italy; nicoletta.medaina@aovr.veneto.it (N.M.); giona.turri@aovr.veneto.it (G.T.)

**Keywords:** NGS, epidemiological surveillance, SARS-CoV-2, variant, lineages, genome evolution

## Abstract

The availability of new technologies for deep sequencing, including next-generation sequencing (NGS), allows for the detection of viral genome variations. The epidemiological determination of SARS-CoV-2 viral genome changes during the pandemic waves displayed the genome evolution and subsequent onset of variants over time. These variants were often associated with a different impact on viral transmission and disease severity. We investigated, in a retrospective study, the trend of SARS-CoV-2-positive samples collected from the start of the Italian pandemic (January 2020) to June 2023. In addition, viral RNAs extracted from 938 nasopharyngeal swab samples were analyzed using NGS between February 2022 and June 2023. Sequences were analyzed with bioinformatic tools to identify lineages and mutations and for phylogenetic studies. Six pandemic waves were detected. In our samples, we predominantly detected BA.2, BQ.1, BA.5.1, BA.5.2, and, more recently, XBB.1 and its subvariants. The data describe the SARS-CoV-2 genome evolution involved in viral interactions with the host and the dynamics of specific genome mutations and deletions.

## 1. Introduction

Severe acute respiratory syndrome coronavirus 2 (SARS-CoV-2) is the etiologic agent of the COVID-19 pandemic. SARS-CoV-2 is classified as being in the *Betacoronavirus* genus of the *Coronaviridae* family [1], and its genome is represented by a single-stranded positive-sense RNA genome of approximately 30 kb, characterized by several functional open reading frames (ORFs), including the replicase (ORF1a/ORF1b), spike (S), envelope (E), membrane (M), and nucleocapsid (N) genes. Coronaviruses are prone to genomic mutations and gene recombination events [2,3]. These characteristics play a pivotal role in overcoming the selective pressures of the environment and the host immune system with a progressive presentation of new viral variants to support viral spread [4]. The sequencing analysis of SARS-CoV-2 genomes isolated during successive pandemic waves demonstrated that viral strains could recombine with each other to create new viral variants, including, for example, XE, XBF, and, more recently, XBB and its subvariants [5]. 

Such variants arise from co-infections with different SARS-CoV-2 lineages and elicit the emergence of new viruses that may impact the pathogenesis and transmission of SARS-CoV-2 [6]. For example, the first mutation discovered in the Spike protein, D614G, was responsible for the increased binding affinity for ACE2 [7] while the mutation L455S seems to increase immune evasion in the recently appeared variant JN.1 while decreasing its ACE2 binding affinity [8]. On the other hand, recombination also plays a pivotal role in the rise of new variants, as in the case of the XBB variant (which emerged following recombination between BA.2.10.1 and BA.2.75 sublineages) [9].

Analyzing World Health Organization (WHO) statistics, we can identify six waves of COVID-19 in Italy: the first in March–April 2020, the second in October–November 2020, the third in February–March 2021, the fourth between December 2021 and January 2022, the fifth in March 2022, and the sixth in October 2022. The variant sequences of SARS-CoV-2 have consistently changed, thus inducing the failure of antibody treatments [10] and/or decreasing vaccine effectiveness [11]. These different waves were related to new variants, in particular, those generated during chronic infection in immunocompromised patients in whom, for example, the Omicron variant was first detected [12]. The development of SARS-CoV-2 whole-genome sequencing protocols, as well as databases enabling access to sequences from around the world [13], allowed for rapid and effective epidemiological surveillance. In this study, we analyzed SARS-CoV-2 incidence between February 2022 to June 2023, investigating the emergence and progression of the different lineages through next-generation sequencing (NGS) routine surveillance over the last two waves of the pandemic, one in July 2022 and one between October and November 2022.

## 2. Materials and Methods

### 2.1. Sample Collection and RNA Extraction and Quantification

Nasopharyngeal swab samples collected from patients and medical surveillance personnel at the AOUI Hospital of Verona as previously described [14] were processed for SARS-CoV-2 infection diagnosis. RNA extraction from nasopharyngeal swab maintenance solution was performed with the semi-automated Nimbus apparatus (Seegene, Seoul, Republic of Korea) following the manufacturer’s instructions. Multiplex RT-PCR was performed with a Bio-Rad CFX96 system (Bio-Rad, Hercules, CA, USA), using the Allplex SARS-CoV-2 Assay (Seegene, Seoul, Republic of Korea). To perform SARS-CoV-2 sequencing, we newly extracted viral RNA from the residual volume of nasopharyngeal swab samples by using the same procedure described above. The purified RNA was processed for NGS analysis. All samples were anonymized following Legislative Decree 196/2003 of the Italian Data Protection Code. The viral sequences were uploaded onto the IRIDA-ARIES platform for ICoGen (https://irida.iss.it/irida21-aries/login accessed on 2 February 2024) and then onto the GISAID database (https://gisaid.org/ accessed on 2 February 2024) as requested by the Italian SARS-CoV-2 sequencing network for the epidemiological analysis and surveillance of the Italian SARS-CoV-2 infection.

The study was performed in accordance with the rules established by the Ethics Committees for Clinical Trials of Verona on retrospective studies. According to Italian regulations (law number 52 on 19 May 2022, article 13) for SARS-CoV-2 infection surveillance and in Authorization n. 9/2016 on retrospective studies released by the Italian data protection authority, no specific approval and informed consent were required.

### 2.2. Next-Generation Sequencing 

To avoid suboptimal sequencing results, we selected all samples whose gene cycle thresholds (Ct) were below 32. Library preparation was performed with the QIAseq DIRECT SARS-CoV-2 Kit (QIAGEN, Hilden, Germany) or the Illumina COVIDSeq Assay (Illumina, San Diego, CA, USA) with the ARTIC v4 and v4.1 primer pool. Samples were sequenced with the Illumina MiSeq instrument (Illumina, San Diego, CA, USA) in paired-end mode (2 × 150 bp) with V3 chemistry. 

### 2.3. Bioinformatic Analysis

The sequence analysis was conducted with a custom pipeline using SAMtools v1.18 [15] and Minimap2 v2.17 [16] on the Linux command line. The Pangolin COVID-19 Lineage Assigner [17] and Nextclade tool by Nextstrain [18] were used to identify mutations and lineages. Further sample read distribution controls were performed using the Integrative Genomics Viewer (IGV) tool [19]. A phylogenetic tree was designed by uploading the FASTA sequences of all samples into the UShER tools of the UCSC Genome Browser (https://genome.ucsc.edu/cgi-bin/hgPhyloPlace accessed on 2 February 2024). Epidemiological analysis on SARS-CoV-2 variants was performed with data obtained from the covSPECTRUM web tool (https://cov-spectrum.org accessed on 2 February 2024).

## 3. Results

### 3.1. Epidemiological Analysis of SARS-CoV-2 Infection

To determine whether the national epidemiological data provided by the WHO were similar to our results, we conducted a retrospective study on the positive SARS-CoV-2 swabs detected at AOUI Verona by comparing the total number of positive cases reported in Italy and in our laboratory each month. It is noteworthy that Verona is placed in a geographical area near the Lombardy region, where the incidence and prevalence were at the highest level in Italy during the pandemic.

**Figure 1:**
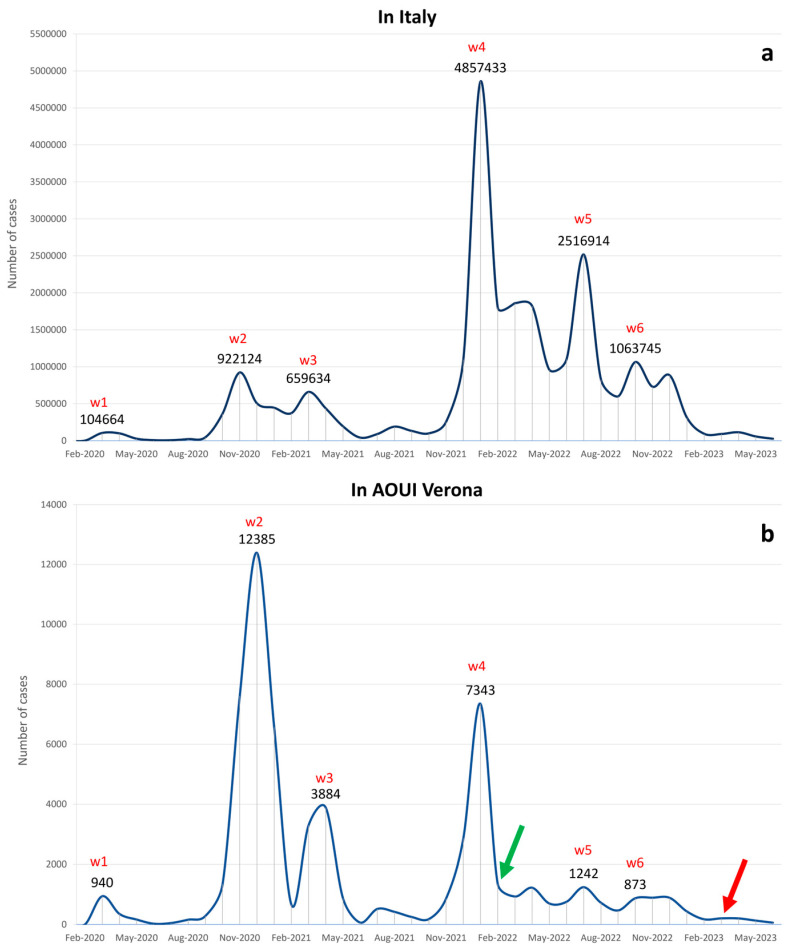
The graph represents the number of positive cases per month from January 2020 to June 2023 in Italy (**a**) and at AOUI Verona (**b**). Peaks identified as epidemiological waves are labeled with the corresponding value. The green arrow on February 2022 indicates the beginning of sequencing screening at AOUI Verona. The red arrow in March 2023 indicates the end of SARS-CoV-2 mandatory surveillance for medical personnel at AOUI Verona. Waves are indicated in red as w1 (wave n° 1), w2 (wave n° 2), w3 (wave n° 3), w4 (wave n° 4), w5 (wave n° 5), and w6 (wave n° 6) above the corresponding peak in the panels (**a**,**b**).

From January 2020 to June 2023, a total of 25,898,945 and 62,329 positive cases of SARS-CoV-2 infection were detected in Italy and at AOUI Verona, respectively. In Figure 1, we report the number of positive cases per month in Italy from the beginning of the pandemic to the end of June 2023 (data provided by the WHO) and the positive cases per month registered at AOUI Verona in the same period (Figure 1a and Figure 1b, respectively). 

In the period corresponding to each epidemiological wave, a peak can be observed in both graphs: the first one in March 2020, the second one in November and December 2020, the third one in April 2021, the fourth wave in January 2022, the fifth wave in July 2022 and the sixth one in October 2022. Regarding the sixth wave, in the AOUI Verona data, there is no clear peak but rather a small plateau, as we reached 892 cases in November 2022 and 887 in December of the same year. The absolute numbers of positive samples for each month are reported in Supplementary Table S1. 

### 3.2. Sequencing of Samples

**Figure 2:**
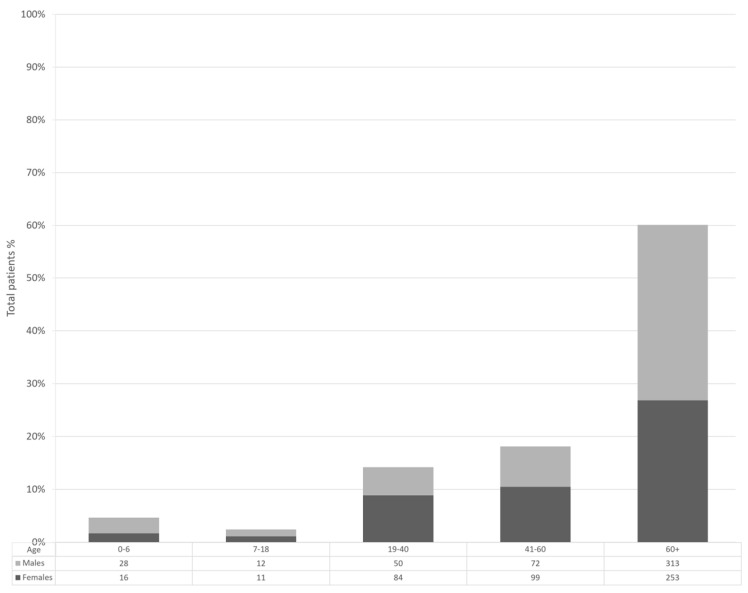
Patient age distribution.

After the epidemiological analysis of the national and local data, we focused on the sequencing data obtained from screening in the local hospital in Verona, which started in February 2022, as represented with a green arrow in Figure 1b. In total, 938 samples were sequenced through NGS and analyzed from February 2022 to June 2023. Fresh samples with a significant viral load (Ct < 32) were processed for library preparation. Samples were selected randomly amongst medical personnel involved in routine surveillance and hospitalized and emergency room patients. Of the selected cases (938 patients), 475 were male and 463 were female (Figure 2).

As expected, the majority of samples (60.34%) were from older patients because of their higher hospitalization. The presence of comorbidities is generally more common in elderly patients, thus creating a bias in randomization.

### 3.3. SARS-CoV-2 Variant Epidemiology

**Figure 3:**
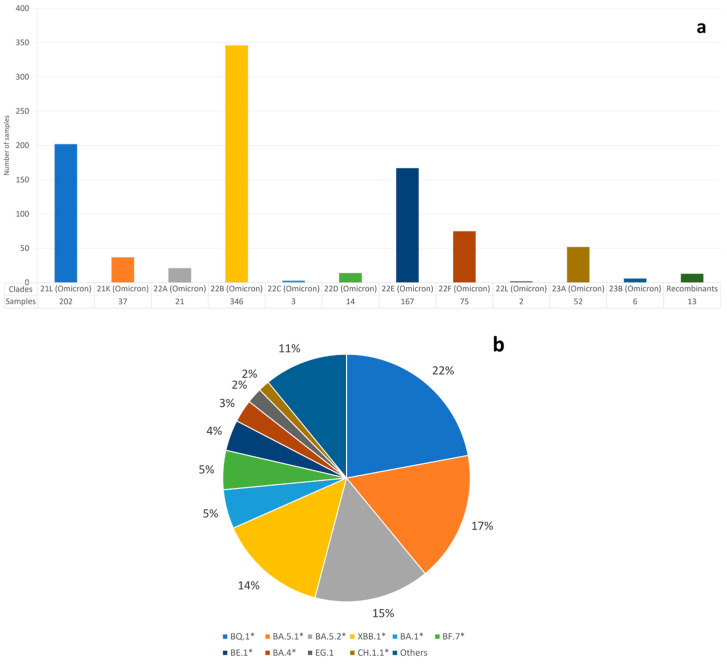
Sample distribution following: (**a**) Nextclade clade and (**b**) Pango lineage criteria. Lineages XE, XBF, XBK, and XAY are all labeled as “Recombinants”. Lineages with * represent the parent lineage and sublineages with the same suffix. Lineages with prevalence < 1% are classified as “Others” according to the Pango Lineage criteria.

We report the sample distribution of variants sequenced in Verona from January 2022 to June 2023 divided by clades (Figure 3a) and grouped by Pango lineage (Figure 3b). XE, XBF, and XBK clades are classified as “Recombinant”. Lineages with * represent the parent lineage and all related sublineages starting with that suffix. BA.5* (clade 22B) and BA.2* (clade 21L and 22C) were the predominant lineages in our dataset, although lineages BQ.1* (clade 22E) and XBB.1* (clade 22F, 23A and 23B) were increasing in prevalence from the beginning of 2023. Lineages indicated as “Others” are lineages that display a final prevalence < 1%. These lineages are BA.5, BA.5.3*, BA.5.5, BA.5.6, BA.5.8, BA.5.9, BE.4*, BF.1, BF.10, BF.11, BF.14, BF.27, BF.28, BF.5, BN.1*, BU.1, CG.1, CK.2.1.1, CL.1, CP.1, DG.1, EG.4, EH.1, EU.1.1.1, FE.1, FL.1.5, FL.2, FL.4, XAY.1.1.1 alias GL.1, XBB, XBB.2*, XBF, XBK, and XE.

During the entire period, the BA.2* lineage showed the greatest prevalence with a total of 207 sequences (22%), followed by BQ.1* (164 sequences, 17%), BA.5.1* and BA.5.2* (126 (13%) and 122 (12%) sequences, respectively), and XBB.1* (106 sequences, 11%). Percentage values below 5% were detected for BA.1*, BF.7*, BE.1*, BA.4*, EG.1*, and CH.1.1* lineages.

It is noteworthy that we submitted to the GISAID some of the earliest sequencing of the XBF and BU.1 variants in Italy. The XBF lineage is a recombinant of BA.5.2.3 and CJ.1 (BA.2.75.3 sublineage) with a global prevalence of 0.17% between February 2022 and June 2023 and reaching peak global incidence (0.63%) between October 2022 and April 2023. The XBF lineage shows a more consistent frequency in Australia (3.42%) in the same period. Interestingly, the XBF Australian wave started in October 2022 and declined in April 2023 with an overall proportion of 9.9%, reaching a peak of 26.17% in the last week of January 2023. In Mexico and Finland, the BU.1 lineage was mainly detected in the first month of 2023 [20], reaching a peak prevalence of 3.89% and 4.67% of the infected populations, respectively. 

The sequence of rise and fall of the observed variant could be explained by a combination of increased binding affinity for ACE2 as in the case of BA.1 and BA.2 [21] and improved immune evasion as in the case of BA.4/5 that was 4.5-fold more resistant than BA.2 to the sera of triple vaccinated patients [22]. Similarly, XBB showed an even higher escape from vaccine-induced antibodies with respect to the BA.5 subvariant BQ.1.1 [23].

In some cases, the variants appeared in Italy a few days after their identification and classification at a global level (such as BF.1, BF.10, BF.28, BF.5, and BQ.1*), while other variants (such as BA.1*, BA.5.6, BE.4*, BF.7*, BN.1*, CH.1.1*, CP.1, XBB.1*, XBB.2.3*, XBF, and XBK) reached Italy later. This could be related to international travel, the reopening of national and international borders, the presence or absence of lockdown periods, the compulsory use of face masks, or, at a viral level, either related to the transmission rate of the specific variant or a low variant prevalence of, for example, XBK.

**Figure 4:**
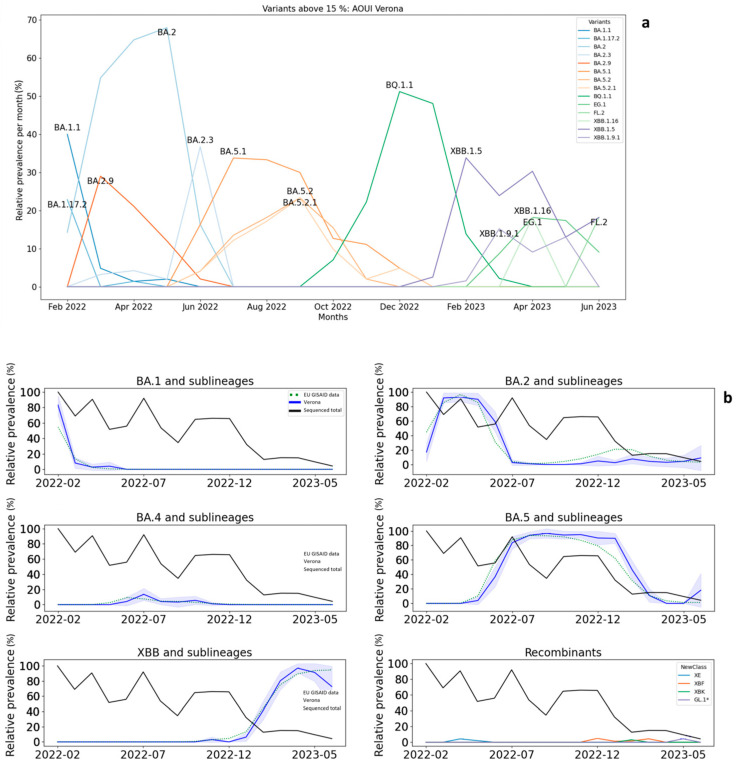
Variant dynamics: (**a**) trend of variants whose prevalence was above 15% during at least one month in the survey; (**b**) changes over time of different variants aggregated according to covSPECTRUM criteria from February 2022 to June 2023. This panel shows dynamics over time of BA.1 and its descendants, BA.2 and its descendants (including BN, BM, CJ, CH, DV, and DS variants), BA.4-related variants (including DC.1), BA.5 and its descendants (including BE, BQ, BF, BU, CL, CG, CP, CK, and EH), and the descendants of the recombinant XBB (including FL, FD, FE, EG, EL, and EU), as well as the dynamics over time of the recombinants (XE, XBF, XBK, and XAY.1.1.1.1 or GL.1). The segmented green lines represent European Union data provided by the GISAID while the continuous blue lines represent our data. The black line indicates the relative local incidence of positive cases at AOUI Verona, taking the maximum incidence as the month with the highest number of positive cases in the investigated period (February). The blue shaded area represents the 95% confidence interval of our data. *: Parent lineage and related sublineages.

To visualize the monthly distribution of the relative prevalence of all variants, excluding variants with a monthly prevalence of below 15%, we constructed a line plot with all of the variants detected at AOUI Verona (Figure 4a). Interestingly, we observed that the BA.1.1, BA.2, BA.2.3, BA.5.1, BQ.1.1, and XBB.1.5 variants each show a prevalence rate in infected patients in Verona of higher than 30%. To further analyze the variant prevalence, we grouped the more frequent variants with their sublineages and created line plots to compare our data with the European data (Figure 4b), also indicating the total incidence of SARS-CoV-2-infected patients in Verona. In particular, the BA.1 variant and its sublineages are related to the fourth pandemic wave, BA.5 is related to the fifth wave, and BQ.1.1 is related to the sixth wave.

**Figure 5:**
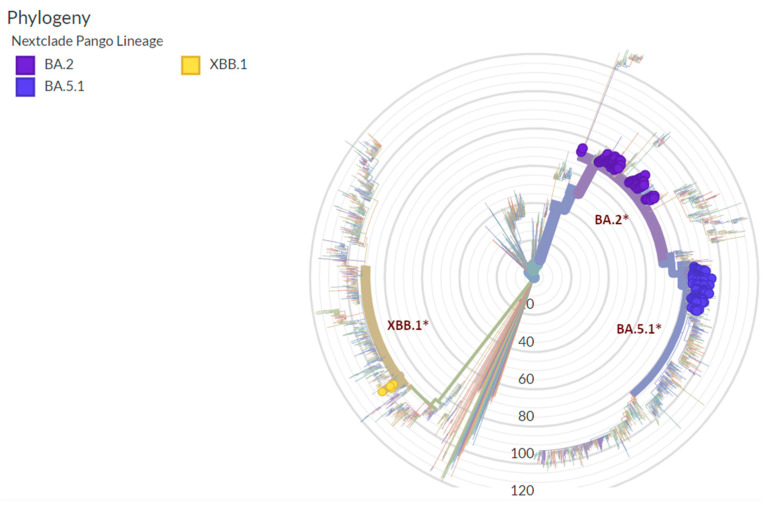
Phylogenetic tree of the lineages BA.2, BA.5.1, and XBB.1 sequences found at AOUI Verona. Representation of the radial tree generated using Nextclade in which branches of BA.2, BA.5.1, XBB.1 and their respective sublineages (*) sequenced samples from AOUI Verona Microbiology unit are highlighted.

Interestingly, variant waves detected in Verona correlate with the European data for all grouped variants and sublineages described in Figure 4b. A phylogenetic tree generated using Nextclade v3.4.0 software (https://clades.nextstrain.org/ accessed on 2 February 2024) of all samples sequenced by the AOUI Verona Microbiology Unit is available in Figure 5, in which the branches with BA.2, BA.5.1, and XBB.1 lineages and their sublineages are highlighted. For a complete phylogenetic tree with all lineages, see Supplementary Figure S1.

## 4. Discussion

Since the onset of the pandemic, SARS-CoV-2 has infected more than 770 million people and caused nearly seven million deaths globally (WHO) up to March 2024. Unlike previous pandemic events, a sequencing approach was able to follow viral transmission, variants, and mutations related to the epidemic waves. 

In this study, we analyzed three years of COVID-19 pandemic data, starting from February 2020 to June 2023 in a specific Italian geographic site represented by the Verona district. In this period, we identified six different waves in which a considerable increase in COVID-19 cases can be observed. We compared reported cases throughout Italy with the positive patients identified in the Microbiology and Virology Laboratory of AOUI Verona, Italy. We observed similar dynamics of incidence development between local and national data but with a greater increase in Verona during the second wave (October–November 2020) compared to the national data in which the wave continued into December. This phenomenon is probably related to the geographical location of Verona, that is, in the Veneto region and at the Lombardy border, two regions where the impact of infection in the first two waves was particularly consistent. Between November and December 2020, after the peak of the second wave, a decrease in numbers can be observed for the national data (from 922,124 positive cases to 505,612). However, from the Istituto Superiore di Sanità (ISS) weekly reports [24], we calculated that the Lombardy region reported over 240,000 cases in November 2020, while the Veneto region reported 98,000 cases; in December of the same year, Lombardy and Veneto reported 73,000 and 120,000 cases, respectively. Intriguingly, we were able to observe a decrease in the number of positive cases nationally and in Lombardy and a rise in numbers in Veneto, where the peak of the second wave was reached in December with 23.74% of the national positive cases for that month. The regional data explain the discrepancy between the national data and the data reported in our laboratory; while the national peak for the second wave was in November 2020, in Veneto, and more specifically in Verona, the number of cases increased until December 2020. We can conclude that, regarding Verona, the second wave occurred from October to December 2020 rather than from October to November.

We also investigated the sequences of 938 samples from different patients of AOUI Verona to determine the variant dynamics. In the entire investigated period, we observed the prevalence of the BA.2* lineage, followed by BQ.1*, BA.5.1*, BA.5.2*, and XBB.1*. These are the most frequent lineages in the period of February 2022 to June 2023. All of the lineages that we observed belong to the Omicron variant of concern that was originally identified in South Africa in November 2021 [20]. No clear signs of co-infection were identified in our samples. Between waves, several lineages were involved in the Italian context. The Alpha variant (B.1.1.7) was predominant until the end of June 2021, with the end of the third wave, then, starting from August, the Delta variant (lineage B.1.617.2) began to increase in frequency until November 2021, exploding in the fourth wave. During this period, more than 99% of the sequenced samples in Italy were referred to as the Delta variant. The last dominant variant, Omicron (B.1.1.529 and its sublineages), first appeared as a major genome in December and remained until the end of the pandemic [24]. A large variety of different lineages belonging to the Omicron variant appeared and disappeared from December 2021 to June 2023. As our NGS screening began in February 2022, we observed the end of BA.1 and the rise of BA.2 between March and April 2022, with an increase in positive cases across the country. However, the fifth wave was dominated by BA.5.1 and BA.5.2 from July until October 2022. The sixth and last wave was characterized by a large array of BQ.1 lineages that carried on until the appearance of XBBs in January 2023, outclassing in a few months the previous variants. Following the epidemiology of the single variants, we can see the downfall of the first major group of lineages of Omicron, BA.1*, which disappeared in March 2022. BA.1 in particular was probably responsible for the last huge increase in the number of new cases during January and the drop in February. The widespread infections with BA.1 were mainly due to the fact that it was three to six times more transmissible than the older variants [25]. Despite this, BA.1 was completely outcompeted by BA.2, a distinct sublineage of Omicron that probably emerged at the same time and differs from the other sublineages as much as the Alpha variant differs from Delta [26,27]. BA.2 had eight more mutations and lacked thirteen with respect to BA.1 [27]. The substitution by variant BA.2* was associated with a brief resurgence in the number of cases but this was significantly lower in respect to the peak in January 2023. The prevalence of BA.2 reached a plateau in April and then decreased until July, when it fell drastically. Subsequently, we observed the appearance of BA.5 and, to a much lesser extent, BA.4, which probably both originated from a common ancestor alongside BA.1 and BA.2 in South Africa in November 2021 and are more closely related to BA.2 [28]. Despite the similarity between the two new variants, BA.5 and its descendants (particularly BQ.1* since September) dominated the following six months and then suddenly vanished. During this long dominance, the number of cases continued to gradually decrease. The recombinant XBB lineages appeared to take over. XBB originated from two BA.2 lineages (BJ.1 and BA.1.75) and was identified in India in August 2022, generating much concern due to the greatly reduced neutralization by antibodies [29]. XBB.1.5 and XBB.1.9 subvariants, in particular, increased in the first months of 2023 and were the more frequent lineage until June 2023. Interestingly, some remnants of both BA.5 and even BA.2 survived until June 2023. The other recombinants were rare: XE, which originated from the crossover between BA.1 and BA.2 [30] appeared briefly in the BA.2 era; XBF, a mixture of BA.5.2.3 and BA.2.75.3 [31], was spotted at the end of the BA.5 dominant phase; XBK, a descendant of both BA.5.2 and CJ.1 [20], also appeared at the very end of the BA.5-prevalent period. Finally, at the end of the present survey, we detected GL.1, a relative of XAY and a complex recombinant between Delta (AY.45) and Omicron (BA.4/5*) [5,20].

Unlike previous pandemic events, a sequencing approach was able to follow viral transmission, variants, and mutations related to the epidemic waves [32].

Regional analysis of SARS-CoV-2 variants and their impact on the number of cases has been carried out before [33]; concerning the Italian case, there was a thorough analysis of the incidence during the first 2 years of the pandemic [34] and also some analysis regarding the relative incidence of Delta and Omicron subvariants [35,36]. However, we went on making a direct comparison with European data in order to understand how local data mirror the wide-scale dynamics of the epidemic.

The temporal accordance between the local and continental datasets is remarkable and demonstrates that new variants spread quickly at the European level and could have implications for how we organize the epidemiological surveillance of future pandemic waves.

During our study, we found six BA.5.1 variant sequences carrying a large (426 nt) deletion spanning ORF7b and ORF8 [37]. This deletion is similar to other deletions that cause the absence of the ORF8-encoded protein. ORF8 is located in a hypervariable genomic region characterized by a high density of hairpins and encodes a protein containing an immunoglobulin-like domain, which undergoes genomic rearrangement [38]. These features facilitated the occurrence of many deletions and mutational events that led to a complete or partial loss of the ORF8 protein during the pandemic era. In SARS-CoV-2 pathogenesis, the role of ORF8 is highlighted by these mutations and their relation to the severity of COVID-19. Due to the hypervariability of the ORF8 coding region and its relation to the viral evolution of SARS-CoV-2, it has been hypothesized that ORF8 plays a key role in driving the virus–host adaptation mechanism [38,39].

We also found a cluster of rare variants, like the XE lineage. As reported previously, XE originated from the recombination of the first part of BA.1, from the beginning of NSP1 to NSP6 genes, and the rest of the genome from BA.2 [40]. The XE lineage had the highest incidence in the UK between February and May 2022, with a total of 2138 sequences (69.46% of the sequences worldwide). In the same period, 42 sequences were found in Italy, with a further cluster of 29 sequences in Campania. In Veneto, second in the number of XE variants found in Italy, a total of six XE were detected, of which four were identified in our laboratory in Verona between April and May 2022. Despite a reported transmissibility 10% greater than its parent BA.2 [41], the XE lineage vanished after this period and was not detected from July 2022 [20].

We have to consider some limitations of this study. At the end of March 2023, mandatory medical surveillance at the AOUI Hospital of Verona, which consisted of screening medical personnel for SARS-CoV-2, was terminated. The drastic reduction in the number of processed samples following the change in surveillance protocol could have impacted the statistical depth of the study. This elicited a decrease in the number of analyzed samples by limiting the tests to hospitalized and emergency room patients. This reduction can be seen clearly in Figure 1, indicated by the red arrow. Moreover, it should be noted that the choice of the sequencing samples is not totally random but is biased by the fact that only samples with low Ct values were effectively sequenced. This technical limitation could not be overcome since a lower Ct value implies a minor viral load and, consequently, suboptimal sequencing quality. Therefore, we sequenced not all but rather a portion of SARS-CoV-2-positive samples, representing the local trend of circulating variants. This trend is consistent with the lineage distribution over time in Europe (Figure 4b).

## 5. Conclusions

Our data show the flow of SARS-CoV-2 during the pandemic, providing valuable insights into the evolution of the SARS-CoV-2 genome and its implications for global public health. By sequencing SARS-CoV-2-positive samples, we have contributed to ongoing surveillance efforts, enabling the monitoring of viral evolution despite our sampling limitations. 

Worldwide, this activity has created a global surveillance and infection monitoring network, converging to important initiatives such as the GISAID. This approach has also determined the analysis of viral evolution and the description of some relatively rare variants, thus demonstrating the complex viral evolution and competition between the different variants due to interaction with the immune response and their infectivity rate. Sequencing can be considered a pivotal tool for epidemiological surveillance to evaluate pandemic evolution and/or specific local infection development.

Our findings highlight the importance of genomic surveillance in understanding pandemic dynamics and guiding public health responses; we have shown that even considering sequencing bias, the local variant trend in a relatively small reality, such as the AOUIVR, mirrors the European trend. It is essential to address remaining questions and challenges in genomic surveillance, including the need for improved sampling strategies and the continued monitoring of viral evolution. By investing in research and surveillance efforts, we can better prepare for future pandemics and protect global health.

## Data Availability

All data from NGS sequencing were available in the GISAID database at https://gisaid.org/ as follows: hCoV-19/Italy/VEN-AOUIVR-*Sample number*_VR/*year* (accessed on 2 February 2024).

## Supplementary Materials

The following supporting information can be downloaded at: https://www.mdpi.com/article/10.3390/microorganisms12050846/s1. Figure S1: Phylogenetic tree of SARS-CoV-2 variants; Table S1: Absolute number of positive samples for each month.
