# Supplementary material for: Surfing the Waves of SARS-CoV-2: Analysis of Viral Genome Variants Using an NGS Survey in Verona, Italy
Source: Microorganisms. 2024 Apr 24;12(5):846. doi: 10.3390/microorganisms12050846 (PMC11124265; doi:10.3390/microorganisms12050846)
Supplement: Supplementary file 1 [file microorganisms-12-00846-s001.zip › Supplementary figure S1.pdf]

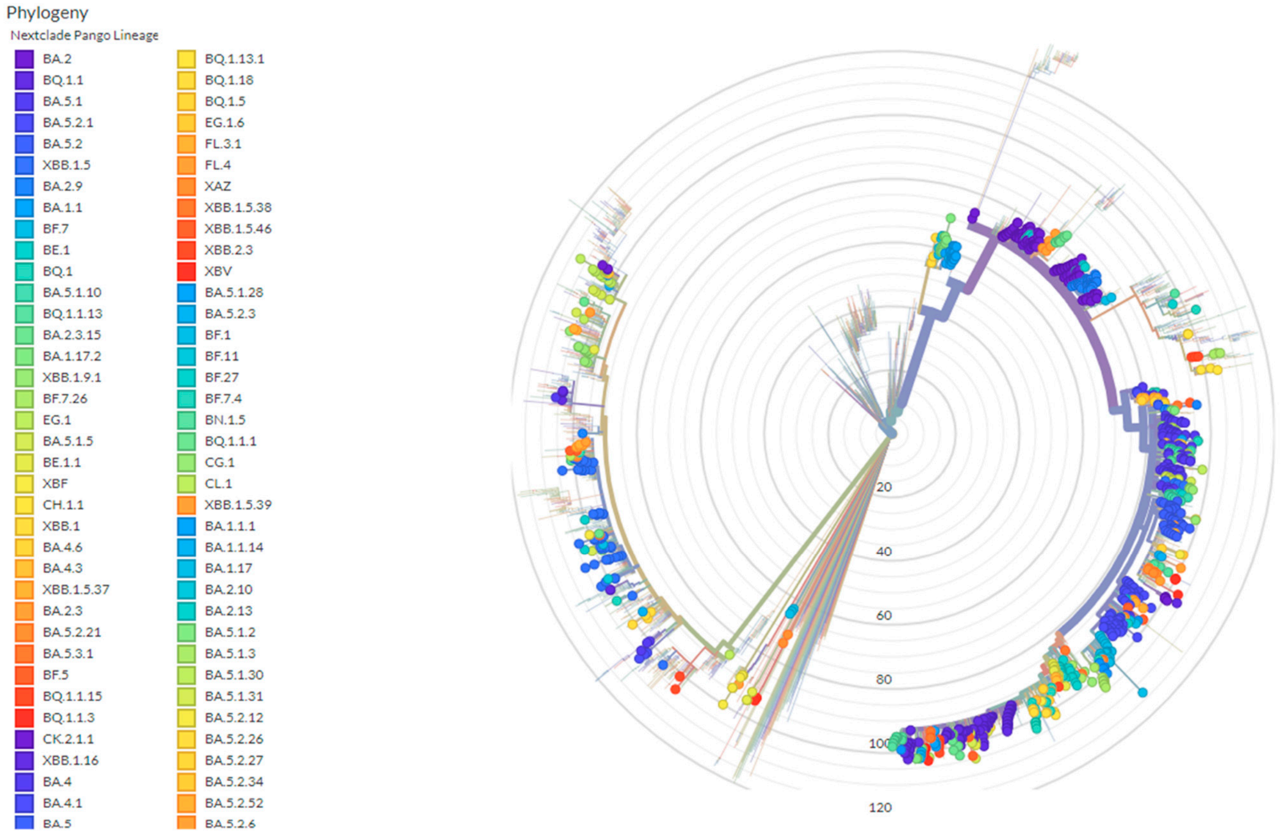

Supplementary Figure S1. *Phylogenetic tree of SARS-CoV-2 variants in AOUI Verona samples.* Representation of the radial tree generated by Nextclade of the 938 sequenced samples from AOUI Verona Microbiology unit. Due to the large number of different Pango lineages identified by Nextclade, the legend is collapsed for a simplified visualization. On request, the EPI_SET ID of GISAID comprehending all sequences can be provided.
