# Supplementary material for: Surfing the Waves of SARS-CoV-2: Analysis of Viral Genome Variants Using an NGS Survey in Verona, Italy
Source: Microorganisms. 2024 Apr 24;12(5):846. doi: 10.3390/microorganisms12050846 (PMC11124265; doi:10.3390/microorganisms12050846)
Supplement: Supplementary file 1 [file microorganisms-12-00846-s001.zip › Supplementary Table S1.pdf]

Year	Month	Positives in Italy	Positives in Verona
2020	January	3	0
	February	1,128	2
	March	104,664	940
	April	99,671	352
	May	27,534	163
	June	7,581	24
	July	6,959	46
	August	21,677	158
	September	45,647	283
	October	364,569	1,347
	November	922,124	7,614
	December	505,612	12,385
2021	January	445,866	6,566
	February	372,233	661
	March	659,634	3,296
	April	437,754	3,884
	May	195,168	874
	June	42,088	62
	July	90,119	517
	August	189,963	419
	September	132,364	248
	October	99,610	180
	November	256,582	868
	December	1,097,136	2,860
2022	January	4,857,433	7,343
	February	1,799,720	1,350
	March	1,859,518	931
	April	1,820,846	1,224
	May	958,210	698
	June	1,101,701	754
	July	2,516,914	1,242

	August	827,732	725
	September	599,521	466
	October	1,063,745	873
	November	729,637	892
	December	883,045	887
2023	January	310,084	433
	February	93,625	172
	March	90,854	203
	April	114,256	201
	May	57,392	128
	June	26,153	58

Supplementary Table S1. Absolute number of positive samples for each month. The table reports the number of positive cases per month from January 2020 to June 2023 in Italy and in Verona.
